# EpistoNet: an ensemble of Epistocracy-optimized mixture of experts for detecting COVID-19 on chest X-ray images

**DOI:** 10.1038/s41598-021-00524-y

**Published:** 2021-11-03

**Authors:** Seyed Ziae Mousavi Mojab, Seyedmohammad Shams, Farshad Fotouhi, Hamid Soltanian-Zadeh

**Affiliations:** 1 Department of Computer Science, College of Engineering, Wayne State University, Detroit, MI USA; 2 Medical Image Analysis Lab, Department of Radiology, Henry Ford Health System, Detroit, MI USA; 3 CIPCE, Department of ECE, College of Engineering, University of Tehran, Tehran, Iran

**Keywords:** Computer science, Computational science

## Abstract

The Coronavirus has spread across the world and infected millions of people, causing devastating damage to the public health and global economies. To mitigate the impact of the coronavirus a reliable, fast, and accurate diagnostic system should be promptly implemented. In this study, we propose EpistoNet, a decision tree-based ensemble model using two mixtures of discriminative experts to classify COVID-19 lung infection from chest X-ray images. To optimize the architecture and hyper-parameters of the designed neural networks, we employed Epistocracy algorithm, a recently proposed hyper-heuristic evolutionary method. Using 2500 chest X-ray images consisting of 1250 COVID-19 and 1250 non-COVID-19 cases, we left out 500 images for testing and partitioned the remaining 2000 images into 5 different clusters using K-means clustering algorithm. We trained multiple deep convolutional neural networks on each cluster to help build a mixture of strong discriminative experts from the top-performing models supervised by a gating network. The final ensemble model obtained 95% accuracy on COVID-19 images and 93% accuracy on non-COVID-19. The experimental results show that EpistoNet can accurately, and reliably be used to detect COVID-19 infection in the chest X-ray images, and Epistocracy algorithm can be effectively used to optimize the hyper-parameters of the proposed models.

## Introduction

The COVID-19 disease caused by severe acute respiratory syndrome coronavirus 2 (SARS-CoV-2) first appeared in Wuhan, China in late December 2019^1^. COVID-19 can infect people of all ages including children and adolescents, resulting in serious complications. According to the World Health Organization, as of January 28, 2021, there have been more than 100 million confirmed cases of COVID-19, including 2,166,440 deaths. This virus can spread in the form of direct contact or by droplets expelled by coughing or sneezing. In some serious cases, COVID-19 can affect the respiratory system and cause severe pneumonia and ultimately death^2^. Pneumonia is a type of infection that causes inflammation in one or both lungs. Currently, a real-time reverse transcription polymerase chain reaction test (rRT-PCR) is required to detect the nucleic acid from SARS-CoV-2 in the respiratory specimens. However, this test is relatively time-consuming, complicated, and generally shows less consistent results^3,4^.

Radiography examination is an alternative screening method used by experienced radiologists to visually diagnose SARS-CoV-2 viral infection. However, the diagnosis of COVID-19 from radiograph images is a massive challenge that require high expertise and dedicated knowledge. According to several studies^5,6^, physical examination of X-ray images by experts provides 70%-80% accuracy. Therefore, a rapid and more accurate diagnosis system that helps physicians screen patients and detect COVID-19 symptoms seems more necessary for an effective and urgent treatment.

The abundant advances in deep learning and digital image processing, specifically in convolution neural networks (CNN), in recent years have opened new possibilities previously thought untenable. Over the past few years, researches developed various computer-aided diagnosis systems (CAD) based on artificial intelligence and machine learning algorithms to detect abnormalities in radiological images such as computed tomography (CT) and X-rays achieving promising results. CT and chest X-ray images (CXR) are generally considered to be fast and effective way for making clinical decisions^7^. Diagnosis of breast cancer^8^, epilepsy^9^, cardiovascular disease^10^, lung cancer^11^, and pneumonia^12^ via deep learning models has become a popular technique in the medical field.

In this paper, we propose a new approach for detecting COVID-19 infection on chest X-ray images using a decision tree-based ensemble model consisting of two mixtures of discriminative experts (MoE) called EpistoNet. The Epistocracy algorithm, a recently proposed hyper-heuristic evolutionary method, has been recruited to build and optimize the neural networks used in this work. The main motivation of developing EpistoNet is to employ it as a diagnostic tool that can help healthcare providers to detect COVID-19 faster, cheaper, and more accurately and accelerate the treatment of those who need it the most. Due to several key differences in other proposed approaches such as the size of the dataset used, the pre-processing steps of the data, statistical noise, hyper-parameter tuning, etc., the highest accuracy we achieved on our testing dataset using other approaches was less than 70%. We decided to develop our own model/algorithms to improve this accuracy. To the best of the authors’ knowledge, there is no similar study that proposed such a model for detecting COVID-19 in chest X-ray images.

The main contributions of this study can be summarized as follows:

1. A new ensemble model called EpistoNet is proposed. EpistoNet is a decision tree-based ensemble model using two mixtures of discriminative experts to classify COVID-19 lung infection from chest X-ray images.
2. A new dataset of 2500 X-ray images is created. All collected images belong to the Henry Ford Health System in Michigan where this research was conducted. These images have been individually reviewed, interpreted, and labeled by experienced radiologists.
3. In order to accurately classify COVID-19 and non-COVID-19 X-ray images, we created a mixture of experts trained on k clusters of visually similar images.
4. We also recruited the Epistocracy algorithm, a recently developed, multi-population, and self-adaptive optimization method to optimize the architecture and hyper-parameters of the designed neural networks.

## Related work

Many researches have been recently proposed methods to detect COVID-19 positive cases from CXR and CT imaging using artificial intelligence (AI) and machine learning (ML) techniques. X-ray images are widely used in the diagnosis and evaluation of various diseases including COVID-19 infections by clinical experts. X-ray radiography is typically less expensive and exposes the patients to much less radiation compared to CT scans^13^. However, clinical diagnosis from X-rays compared to other imaging modalities is much more difficult^14^ and requires significant training and expertise.

El Asnaoui et al.^15^ conducted a comparative study using various deep learning models (VGG16, VGG19, DenseNet201, InceptionResNetV2, InceptionV3, Resnet50, and MobileNetV2) to detect and classify COVID-19.The experiments were performed using 6087 chest X-ray & CT images cases of COVID-19. The dataset was randomly split with 80% of the images for training and 20% for validation. The highest accuracy was achieved by InceptionResNetV2 with 92.18% of overall accuracy and 82.80% accuracy for detecting patients with Coronavirus.

A deep learning-based method called COVID19XrayNet was proposed by Zhang et al.^16^ to predict COVID-19 from X-ray images. COVID19XrayNet comprises of two-step transfer learning pipeline based on ResNet32 with two newly integrated layers: smoothing layer (FSL) and feature extraction layer. COVID19XrayNet achieved 91.92% overall accuracy outperforming the original version of ResNet32.

Hemdan et al.^17^ suggested COVIDX-Net, a deep learning framework based on seven convolutional neural network models namely MobileNetV2, VGG19, InceptionV3, DenseNet201, InceptionResNetV2, ResNetV2 and Xception to detect COVID-19 from chest X-ray images. COVIDX-Net was validated on 50 images comprised of 25 COVID-19 positive cases and 25 normal cases. In their review, VGG19 and DenseNet showed the best results of classification with f1-scores of 91% and 89% for COVID-19 and normal, respectively.

In order to identify COVID-19 from normal or other pneumonia cases, Horry et al.^18^ proposed a multimodal classification network based on optimized VGG19 architecture. Before training their model, they applied histogram equalization to images followed by enhancement to textures and contrasts using OpenCV library. Their proposed network achieved 86% accuracy on X-ray images, 84% for CT scans, and 100% for Ultrasound.

In Wang et al.^3^, the authors presented COVID-Net, a deep convolutional neural network consisting of a heterogeneous mix of convolution layers with variation of kernel sizes for the detection of COVID-19 cases from chest X-rays. COVID-Net was trained and tested on COVIDx dataset comprised of 13,975 chest X-ray images. The proposed model was able to achieve an overall test accuracy of 93.3% and 91% accuracy specifically for COVID-19 cases.

Rahimzadeh et al.^19^ proposed deep convolution network based on the concatenation of Xception and ReNet50V2. They evaluated their model on 11,302 chest X-ray images, consisting of only 31 cases of COVID-19 and 11,271 cases from the other two classes. Their proposed model achieved an average accuracy of 99.50%, and 80.53% sensitivity for the COVID-19, and an overall accuracy of 91.4%.

Kaur et al.^20^ proposed a metaheuristic-based deep COVID-19 screening model using modified AlexNet architecture for feature extraction and classification of the input images. Strength Pareto evolutionary algorithm-II (SPEA-II) was used to tune the hyper-parameters of modified AlexNet. The proposed model achieved a validation accuracy of 99.26%.

COVID-CheXNet, is another hybrid deep learning framework developed by Al-Waisy et al.^21^ to diagnose COVID-19 infection from the X-ray images. The COVID-CheXNet system combines the results obtained from two different pre-trained deep learning models based on ResNet34 and HRNet (high-resolution network model) trained using a large-scale dataset. By enhancing the contrast of the X-ray images and reducing the noise level using the contrast-limited adaptive histogram equalization and Butterworth bandpass filter, the proposed COVID-CheXNet system has managed to diagnose the COVID-19 patients with a detection accuracy rate of 99.99%.

Moreover, Mohammed et al.^22^ did a comprehensive investigation of ML based classification methods for automated diagnosis of COVID-19. Based on the results obtained from different experiments, ResNet50 model had achieved the optimum accuracy of 98.8% while the traditional techniques such as SVM demonstrated the best result for an accuracy of 95% and RBF (Radial basis function) an accuracy of 94% for the prediction of coronavirus disease.

Finally, Ismael et al.^23^ reported another deep learning approach that allows detection of COVID-19 patients. Authors used pretrained deep CNN models (ResNet18, ResNet50, ResNet101, VGG16, and VGG19) for feature extraction, and the Support Vector Machines (SVM) for classification. Their dataset contained 180 COVID-19 and 200 normal chest X-ray images. The deep features extracted from the ResNet50 model and SVM classifier achieved an accuracy of 94.7%.

These approaches lack the generalizability for unseen data due to various pre-processing steps performed and assumptions involved in the model development and hyper-parameter fine tuning conducted specific to their own dataset. In this paper we describe the development and evaluation of a new approach for detection of COVID-19 from chest X-ray images using a minimal pre-processing pipeline and automatic optimization of the hyper-parameters of various models using a recently proposed algorithm.

## Methods

In this section, we will discuss the architecture design methodology and the key components of EpistoNet, motivated by the need of developing a feasible solution to help combat COVID-19.

**Figure 1:**
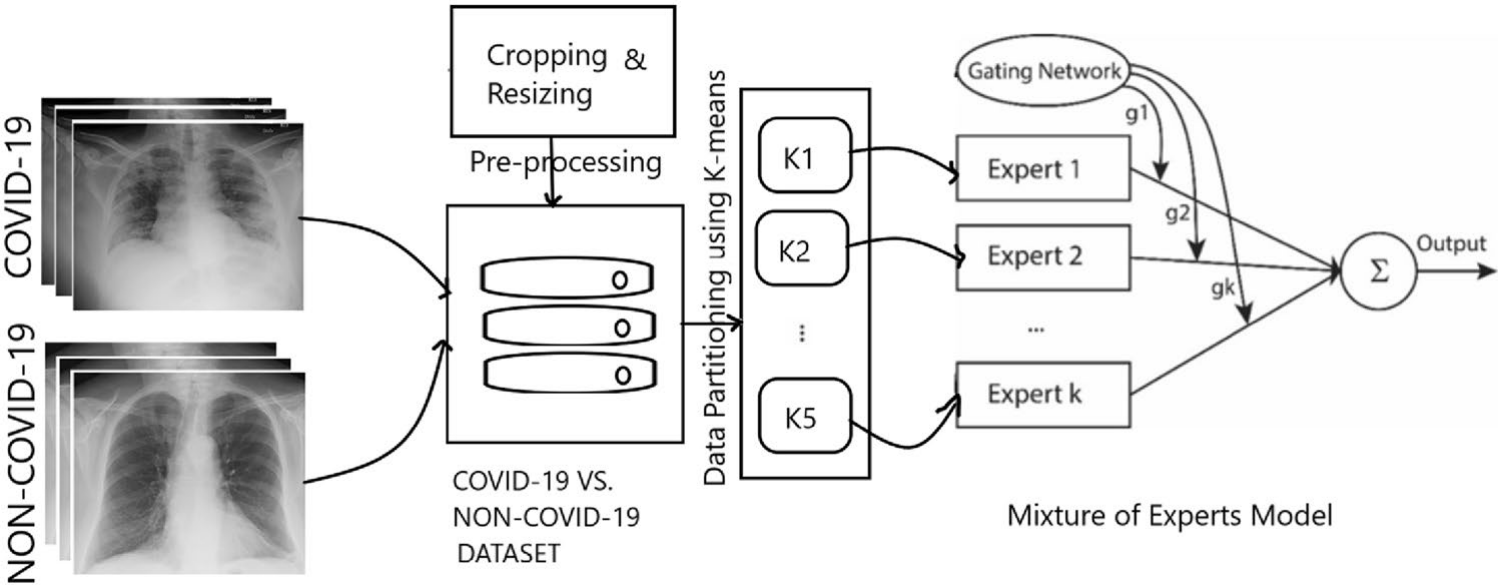
Block diagram of the proposed method for diagnosing COVID-19 in chest X-rays images.

As depicted in Fig. 1, first, the procedure to create the training dataset was briefly described. Then, the pre-processing steps, the EpistoNet architecture design, and the optimization of expert networks of the proposed approach were explained.

### Dataset description

**Figure 2:**
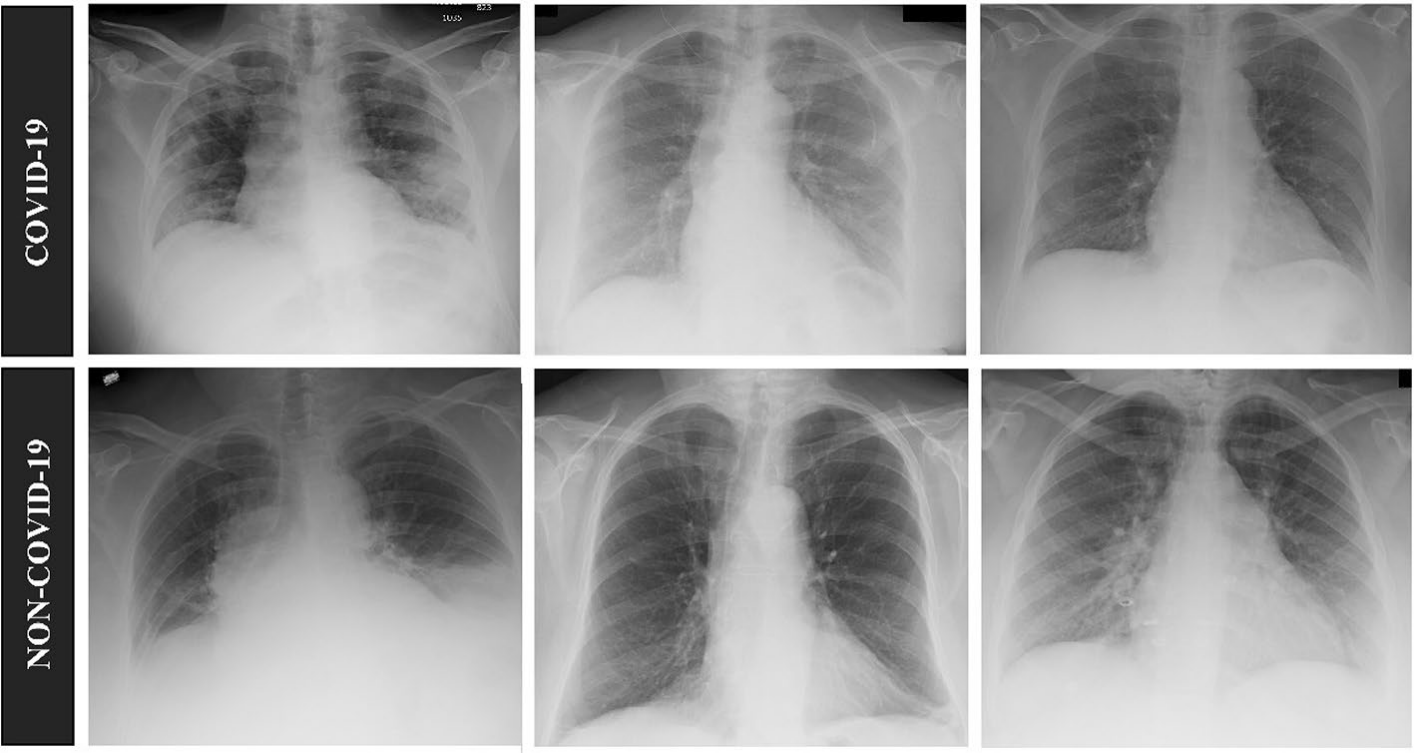
Examples of COVID-19 and non-COVID-19 chest X-ray images.

The dataset utilized in this research is comprised of 2500 X-ray images consisting of 1250 COVID-19 and 1250 non-COVID-19 images provided by Henry Ford Health System (HFHS) of Michigan in Detroit (see Table 1). All images stored in JPEG format containing 3 channels of 8-bit data. The non-COVID-19 images include normal, as well as non-COVID-19 viral and bacterial pneumonia infections (see Fig. 2). These X-rays depict the front view of a patient’s upper torso, with a clear view of the lungs. All images have been cropped to frame the entire rib cage with reasonable padding space and down sampled to 224 by 224 pixels when compiled into a singular dataset. No other modification or image enhancement was done to the original images to further minimize the time between taking the X-ray and detection of COVID-19. Out of 2500 images, we left out 500 images containing 250 COVID-19 and 250 non-COVID-19 for testing. The remaining 2000 images were used 80% for training and 20% for validation.

**Table 1:** Distribution of X-ray images in training, validation, and testing datasets.

Dataset	COVID-19	Non-COVID-19	Total
Training	800	800	**1600**
Validation	200	200	**400**
Testing	250	250	**500**
Total	**1250**	**1250**	**2500**

The total number of X-ray images in each category and across the dataset is displayed in bold.

Figure 2 shows examples of the X-ray images that we received, with the first set of images representing COVID-19, and the second set representing non-COVID-19 images:

In this study, we did not use online datasets mainly due to the limited number of COVID-19 positive cases available in the public datasets and lack of verification mechanisms that allows us to verify the validity and reliability of these datasets.

### Ethical issues

First, our study used anonymized X-ray images collected at the Department of Radiology, Henry Ford Health System (HFHS), Detroit, MI. There were no potentially identifying marks/features and no patient identifiers in the images. This study was approved by the IRB committee of HFHS (No. 14030).

Secondly, the IRB committee of HFHS waived the need for obtaining the informed consent for this study.

Thirdly, all methods were performed in accordance with the relevant guidelines and regulations, including those of the Declaration of Helsinki.

### EpistoNet architecture design

To build an efficient classification model, we propose a method using mixture of deep CNN experts to detect COVID-19 from chest X-ray images. The identification and extraction of relevant features from X-ray images is a challenging task that requires multiple neural network architectures to directly operate on the given data and find patterns that help in detection and classification of the COVID-19 infection. For this purpose, we have designed an ensemble model which is able to exploit discriminative features and obtain higher accuracy than individual CNN models on the HFHS dataset.

#### Mixture of experts model

Mixture of experts is a type of ensemble based on the divide-and-conquer principle where each individual model is specialized in a given part of the input space, learning different aspect of the problem.

**Figure 3:**
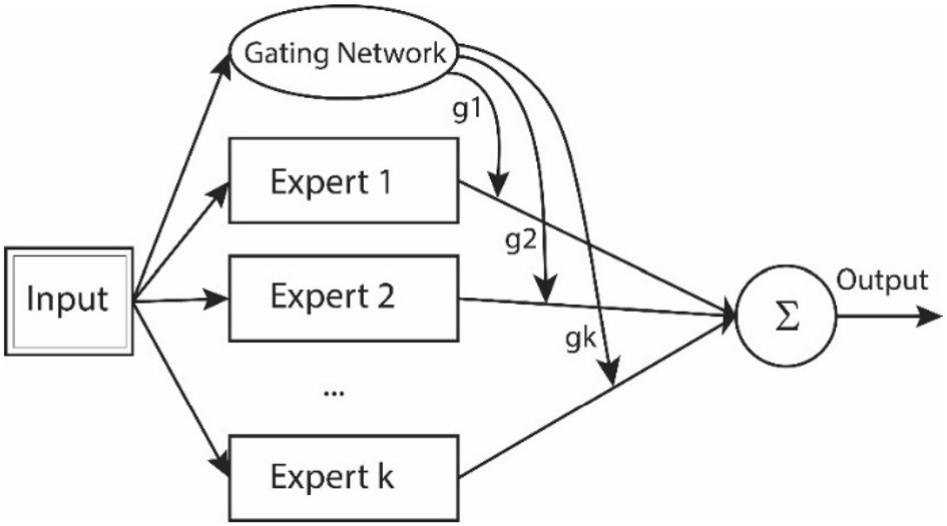
Mixture of experts model.

As shown in Fig. 3, MoE architecture is composed of *k* expert models which are supervised by a gating network. The gating network is a discriminator network trained together with the experts on the same input and decides which expert(s) to use for the final classification task.

1 $$y\left( x \right) = \sum\limits_{i = 1}^{k} {g_{i} } \left( x \right)y_{i} \left( x \right)$$

The output of the gating network can be interpreted as the probability that input *x* is assigned to expert *i* (see Eq. (1)). The gating network employs softmax function for activation:

2 $$g_{i} = \frac{{e^{{z_{i} }} }}{{\sum\nolimits_{j = 1}^{k} {e^{{z_{j} }} } }}$$

in Eq. (2), $z_{j}$ is the output of the gating network. The softmax function makes the outputs of the gating network sum to one. This network of experts can potentially improve the accuracy and the reliability of the overall classification system^24^.

#### Data partitioning using K-means clustering method

In order to effectively discriminate COVID-19 from non-COVID-19 X-ray images, we decided to split our main dataset into *k* clusters and train explicitly localized expert networks on each cluster capable of differentiating between visually similar images. To this point, first we applied a cluster-based pre-processing step to our dataset of 2000 images and partitioned them into 5 clusters of variable size using K-means clustering method. K-means is a type of unsupervised machine learning technique commonly used for clustering unlabeled data into *k* clusters.

#### Optimization of expert networks using Epistocracy algorithm

**Figure 4:**
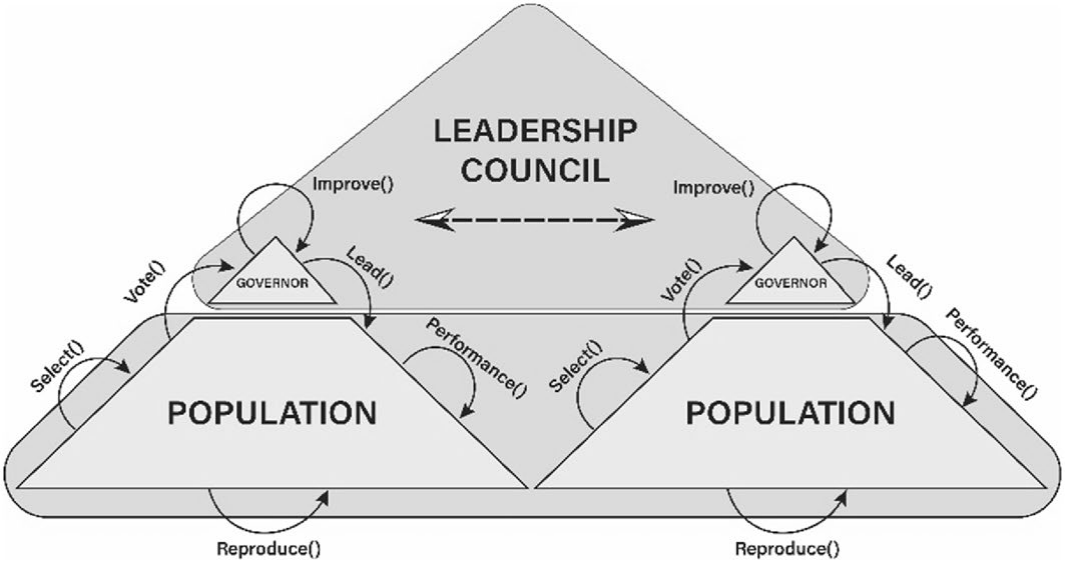
Flow diagram of Epistocracy algorithm.

To further improve the accuracy of each convolutional neural network, we have used Epistocracy algorithm^25^. Epistocracy algorithm is a multi-population self-adaptive optimization method that uses different explorative and exploitative techniques to search the problem space and find the optimal solution. To avoid stagnation and to prevent a premature convergence, the algorithm employs multiple mechanisms such as dynamic population allocation and regression-based leadership adjustment. The algorithm uses a stratified sampling method called Latin Hypercube Sampling (LHS)^26^ to evenly distribute the initial population for an efficient exploration of the search space. Figure 4 shows the flow diagram of the proposed algorithm.

As illustrated in Fig. 4, the Epistocracy algorithm is made of two key components: Governors and Citizens. Citizens are individual solutions that are randomly, and uniformly generated. In each iteration, all individuals are evaluated with a pre-defined fitness function. Governors are the top-performing individuals who are selected through the Select() function to lead the population and influence and evolve the generation of the new population via Lead() function. In Epistocracy algorithm, citizens can directly vote for governors and affect their position in the government.

The architecture of each expert model is made of a base and a head. The base model is a popular CNN model pre-trained on ImageNet for transfer learning. The head model which consists of fully connected layers, is automatically constructed using Epistocracy algorithm. By repeatedly evolving each architecture and optimizing their corresponding hyper-parameters, Epistocracy algorithm can effectively produce the optimal model fine-tuned for classification.

#### Neural network architecture design

**Figure 5:**
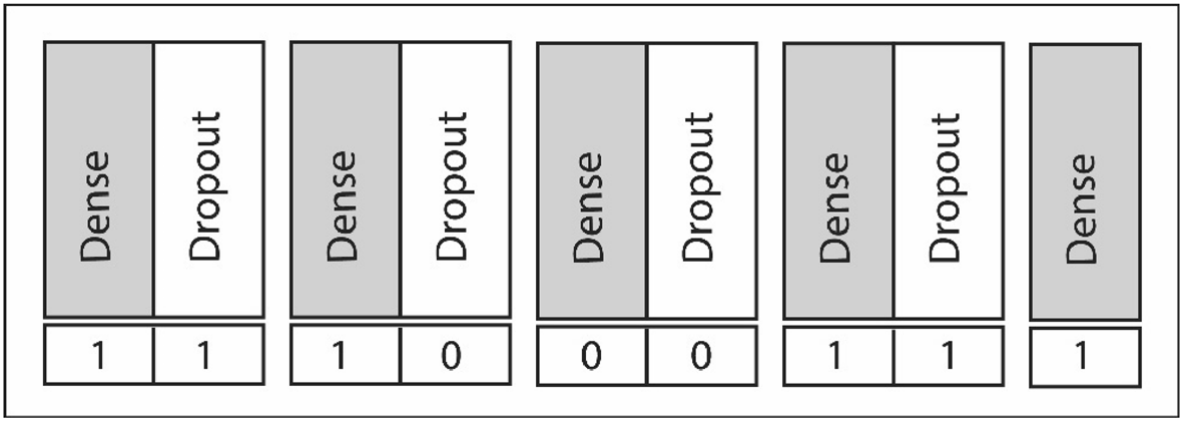
This specific chromosome would result in the following sequence of layers: dense, dropout, dense, dense, dropout, dense (classification layer). The last layer is always on since this classification layer is a required component of the model. Number 1 indicates “on” and 0 indicates “off”.

Using Epistocracy algorithm, the architecture of each neural network in the initial population is generated on a modular basis, in which each module consists of 1 dense layer and 1 dropout layer (see Fig. 5). The MAX_DENSE_LAYERS is used as a variable to define the maximum number of dense layers allowed in the head model. The last module of the architecture only contains 1 dense layer with two neurons performing the binary classification task. Each layer in the fully connected layers are randomly switched on and off with a given probability to randomly create variable length architectures. Figure 5 illustrates a modular example for the fully connected layers.

In addition to generating the architecture of neural networks, the hyperparameters of the fully connected layers are also individually randomized. These hyperparameters are randomly selected to increase the diversity of the population and the possibility of finding an optimal one. The number of neurons, the activation method, and the dropout rate are randomly selected from Table 2:

**Table 2:** Hyper-parameters of the dense and dropout layers.

Hyper-parameter	Value
Number of neurons	7, 8, 9, 16, 32, 64, 128, 256, 512, 1024, 2048, 4096
Activation method	‘relu’, ‘elu’, ‘sigmoid’, ‘softmax’, ‘tanh’
Dropout rate	0.1, 0.2, 0.3, 0.4, 0.5

The Epistocracy algorithm strives to find the optimal CNN architecture in an efficient amount of time.

#### CNN fitness score function

A model is first created by calling a unique function specific to the desired CNN architecture such as VGG16. This function takes the mixed list of hyper-parameter values and returns a model built with those values. Included within this function is functionality to map each hyper-parameter value to its respective place in the layer construction of the model. The new model is then trained on the input dataset using global variables for training parameters (such as number of epochs, etc.). Once training is complete, the validation accuracy score for the model is retrieved from the training history and returned by the function.

#### Epistocracy parameters

When running Epistocracy, the population size (number of individuals) was set to 100. Mutation rate was 20%, and crossover rate (number of individuals recombined genetically each generation) was 80%. 20 full generations of Epistocracy were run in full without any early stoppage. After Epistocracy fully runs and various architectures are generated the top performing one is finally returned.

#### The proposed architecture of EpistoNet

**Figure 6:**
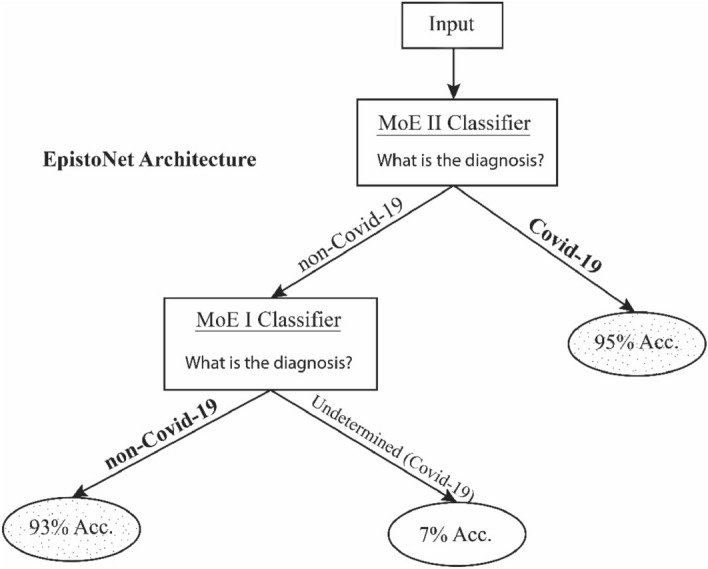
The decision tree of EpistoNet, including two mixtures of experts.

The proposed EpistoNet decision tree is then designed using MoE I and MoE II as shown in Fig. 6:

## Experimental results and discussion

Extensive experiments were performed to evaluate the performance of the proposed model to classify COVID-19 from chest X-ray images. In our experiment, we set the training, and validation ratios to 80%, and 20% respectively. We hold out 25% of the entire dataset, namely 500 images out of 2500 for testing.

### Evaluation of deep convolutional neural networks for detection of COVID-19

To identify the best classification model, we employed deep convolutional neural networks of different depth and complexity and evaluated their performance using HFHS testing dataset. We applied transfer learning to initialize the data training and to facilitate the feature extraction from the input data using ImageNet weights. As it is shown in Table 3, VGG16 achieved the highest accuracy (0.86%) among eight different CNN models. This is treated as ground truth for comparing the accuracy of the proposed model.

**Table 3:** Classification accuracy of each CNN individual model on HFHS testing dataset.

CNN model	Accuracy	Classes	Precision	Recall	F1-score
VGG16	**0.86**	Non-COVID	0.88	0.84	**0.86**
		COVID	0.85	0.88	**0.86**
VGG19	0.85	Non-COVID	0.92	0.76	0.83
		COVID	0.80	0.94	0.86
Xception	0.80	Non-COVID	0.78	0.83	0.80
		COVID	0.82	0.76	0.79
InceptionV3	0.79	Non-COVID	0.81	0.76	0.79
		COVID	0.78	0.82	0.80
InceptionResNetV2	0.86	Non-COVID	0.85	0.87	0.86
		COVID	0.86	0.84	0.85
ResNet50V2	0.82	Non-COVID	0.84	0.79	0.81
		COVID	0.80	0.85	0.82
EfficientNetB7	0.50	Non-COVID	0.50	1.00	0.67
		COVID	0.00	0.00	0.00
MobileNetV2	0.84	Non-COVID	0.85	0.82	0.84
		COVID	0.83	0.86	0.84

The highest accuracy obtained is displayed in bold.

### Evaluation of cluster-based CNN models

**Figure 7:**
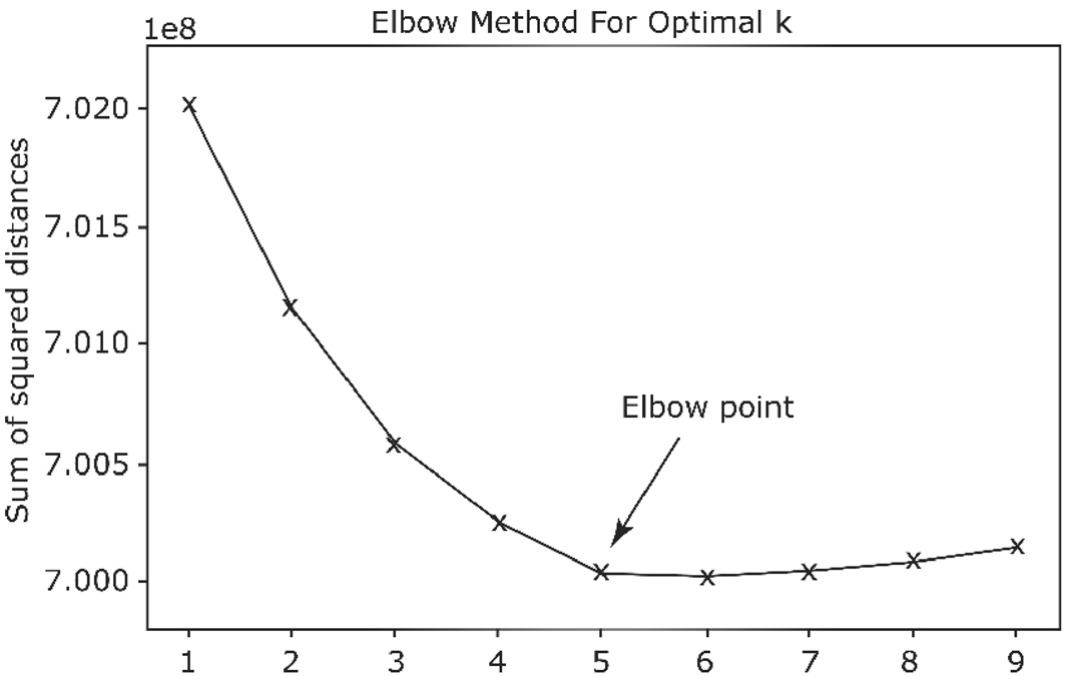
The elbow method used to estimate the optimal value for K = 5.

To determine the optimal number of clusters we employed the elbow method. In fact, we tried different numbers of clusters *k*, and plotted the number of clusters *k* versus the inertia which is the average of the squared distances from the cluster centers of the respective clusters. As it is shown in Fig. 7, for the given data, the optimal number of clusters is 5, where inertia starts decreasing in a linear fashion.

To design our classifier, we employed eight state-of-the-art CNN models on each cluster (see Table 4). To train each CNN model, we used 50 epochs and a batch size of 32. The input data was split into training and validation, with 80% of the data used for training and 20% for validation. The results for each trained CNN are illustrated in Table 4.

**Table 4:** Classification accuracy of different CNN models trained on each cluster.

Clusters			M1	M2	M3	M4	M5	M6	M7	M8
C0	**Accuracy**		0.90	0.90	0.85	0.88	**0.93**	0.80	0.62	0.85
	Precision	Non-COVID	0.86	0.89	0.83	0.95	0.92	0.76	0.62	0.85
		COVID	1.00	0.92	0.91	0.78	0.93	1.00	0.00	0.85
	Recall	Non-COVID	1.00	0.96	0.96	0.84	0.96	1.00	1.00	0.92
		COVID	0.73	0.80	0.67	0.93	0.87	0.47	0.00	0.73
	F1-score	Non-COVID	0.93	0.92	0.89	0.89	0.94	0.86	0.77	0.88
		COVID	0.85	0.86	0.77	0.85	0.90	0.64	0.00	0.79
C1	**Accuracy**		0.81	**0.85**	0.73	0.75	0.77	0.82	0.75	0.81
	Precision	Non-COVID	0.85	0.87	0.80	0.79	0.83	0.82	0.75	0.86
		COVID	0.64	0.77	0.43	0.50	0.53	0.86	0.00	0.62
	Recall	Non-COVID	0.91	0.95	0.85	0.91	0.87	0.98	1.00	0.89
		COVID	0.50	0.56	0.33	0.28	0.44	0.33	0.00	0.56
	F1-score	Non-COVID	0.88	0.90	0.82	0.85	0.85	0.89	0.86	0.88
		COVID	0.56	0.65	0.38	0.36	0.48	0.48	0.00	0.59
C2	**Accuracy**		0.87	0.80	0.82	0.86	0.83	0.86	0.60	**0.87**
	Precision	Non-COVID	0.86	0.77	0.84	0.86	0.84	0.83	0.60	0.87
		COVID	0.90	0.88	0.78	0.85	0.80	0.92	0.00	0.89
	Recall	Non-COVID	0.95	0.95	0.86	0.91	0.88	0.96	1.00	0.93
		COVID	0.76	0.58	0.76	0.78	0.74	0.70	0.00	0.78
	F1-score	Non-COVID	0.90	0.85	0.85	0.88	0.86	0.89	0.75	0.90
		COVID	0.83	0.70	0.77	0.81	0.77	0.80	0.00	0.83
C3	**Accuracy**		0.78	0.84	0.73	0.81	**0.87**	0.84	0.68	0.80
	Precision	Non-COVID	0.67	0.81	0.58	0.76	0.84	0.77	0.00	0.77
		COVID	0.82	0.86	0.80	0.83	0.88	0.88	0.68	0.81
	Recall	Non-COVID	0.59	0.68	0.57	0.59	0.73	0.73	0.00	0.54
		COVID	0.86	0.92	0.81	0.91	0.94	0.90	1.00	0.92
	F1-score	Non-COVID	0.63	0.74	0.58	0.67	0.78	0.75	0.00	0.63
		COVID	0.84	0.89	0.81	0.87	0.90	0.89	0.81	0.86
C4	**Accuracy**		0.91	**0.93**	0.83	0.87	0.83	0.85	0.85	0.89
	Precision	Non-COVID	0.80	1.00	0.43	1.00	0.00	0.00	0.00	1.00
		COVID	0.93	0.93	0.90	0.87	0.85	0.85	0.85	0.89
	Recall	Non-COVID	0.57	0.57	0.43	0.14	0.00	0.00	0.00	0.29
		COVID	0.97	1.00	0.90	1.00	0.97	1.00	1.00	1.00
	F1-score	Non-COVID	0.67	0.73	0.43	0.25	0.00	0.00	0.00	0.44
		COVID	0.95	0.96	0.90	0.93	0.91	0.92	0.92	0.94

The highest classification accuracies among eight different CNN models are marked in bold.

In Table 4, M1–M8 models are VGG16, VGG19, Xception, InceptionV3, InceptionResNetV2, ResNet50V2, EfficientNetB7, and MobileNetV2 respectively.

### Optimization and fine-tuning of CNN parameters

To further optimize the performance of CNN models, we applied the Epistocracy algorithm to generate the optimal architecture of the neural network classifier. As shown in Table 5, the CNN models were noticeably improved, confirming the capability of Epistocracy algorithm in the optimization of complex and non-linear problems.

**Table 5:** Improving the accuracy of expert models using Epistocracy algorithm.

Expert	Model	Accuracy	Classes	Precision	Recall	F1-score
C0	Original InceptionResNetV2	0.93	Non-COVID	0.92	0.96	0.94
			COVID	0.93	0.87	0.90
	Epistocracy optimized InceptionResNetV2	**0.95**	Non-COVID	0.96	0.96	0.96
			COVID	0.93	0.93	0.93
C1	Original VGG19	0.85	Non-COVID	0.87	0.95	0.90
			COVID	0.77	0.56	0.65
	Epistocracy optimized VGG19	**0.88**	Non-COVID	0.91	0.93	0.92
			COVID	0.76	0.72	0.74
C2	Original MobileNetV2	0.87	Non-COVID	0.87	0.93	0.90
			COVID	0.89	0.78	0.83
	Epistocracy optimized MobileNetV2	**0.90**	Non-COVID	0.92	0.92	0.92
			COVID	0.88	0.88	0.88
C3	Original InceptionResNetV2	0.87	Non-COVID	0.84	0.73	0.78
			COVID	0.88	0.94	0.90
	Epistocracy optimized InceptionResNetV2	**0.88**	Non-COVID	0.90	0.70	0.79
			COVID	0.87	0.96	0.92
C4	Original VGG19	0.93	Non-COVID	1.00	0.57	0.73
			COVID	0.93	1.00	0.96
	Epistocracy optimized VGG19	**0.94**	Non-COVID	0.75	0.86	0.80
			COVID	0.97	0.95	0.96

The numbers in bold represent the improved accuracy after using Epistocracy algorithm.

Next, we designed a mixture of experts consisting of 5 optimized CNN models. To choose a gating network for the mixture of experts, we trained and tested different CNN models. From the experimental results obtained (see Table 6), InceptionV3 achieved the highest accuracy for detection of COVID-19 cases among all CNN models, whereas InceptionResNetV2 presented the highest accuracy for non-COVID-19 cases. To improve the performance of the classification model, therefore, we built two mixtures of experts with different gating networks. In the first mixture of experts (MoE I), we used InceptionV3, and in the second mixture of experts (MoE II) we used InceptionResNetV2.

**Table 6:** Classification accuracy of the entire MoE using different CNN models to serve as a gating network.

Gating network	Val. acc.	Testing acc.	Classes	Precision	Recall	F1-score
VGG16	0.91	0.83	Non-COVID	0.82	0.85	0.83
			COVID	0.84	0.81	0.83
VGG19	0.9225	0.85	Non-COVID	0.84	0.87	0.85
			COVID	0.87	0.83	0.85
Xception	0.9250	0.87	Non-COVID	0.88	0.85	0.86
			COVID	0.86	0.88	0.87
InceptionV3	0.9225	0.90	Non-COVID	0.88	**0.91**	0.90
			COVID	0.91	0.88	0.89
InceptionResNetV2	0.9125	0.89	Non-COVID	0.89	0.88	0.88
			COVID	0.88	**0.90**	0.89
MobileNetV2	0.92	0.86	Non-COVID	0.84	0.88	0.86
			COVID	0.88	0.83	0.85
EfficientNetB7	0.9175	0.83	Non-COVID	0.84	0.81	0.83
			COVID	0.82	0.85	0.83
ResNet50V2	0.9125	0.85	Non-COVID	0.85	0.86	0.85
			COVID	0.86	0.85	0.85

Precision, Recall, and F1-score are calculated using the testing dataset.

The highest accuracies achieved using different gating networks are marked in bold.

**Figure 8:**
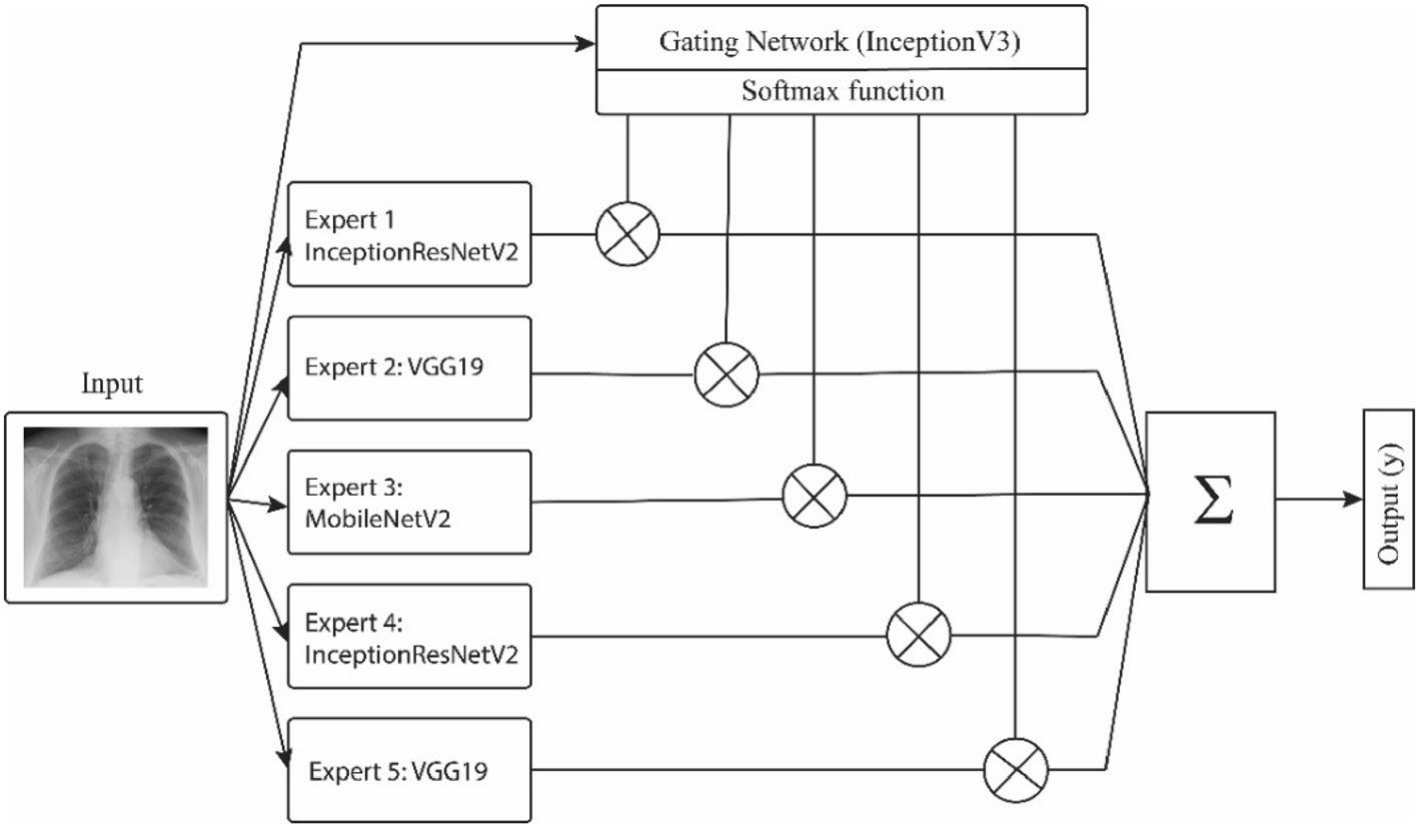
MoE I is a mixture of experts used for classification of COVID-19 infection.

As shown in Fig. 8, we employed 5 expert networks and a gating network to compute the weights for each expert and dynamically combine the inputs. The weights of the gating network are adjusted during the general training of the model on HFHS training dataset.

**Figure 9:**
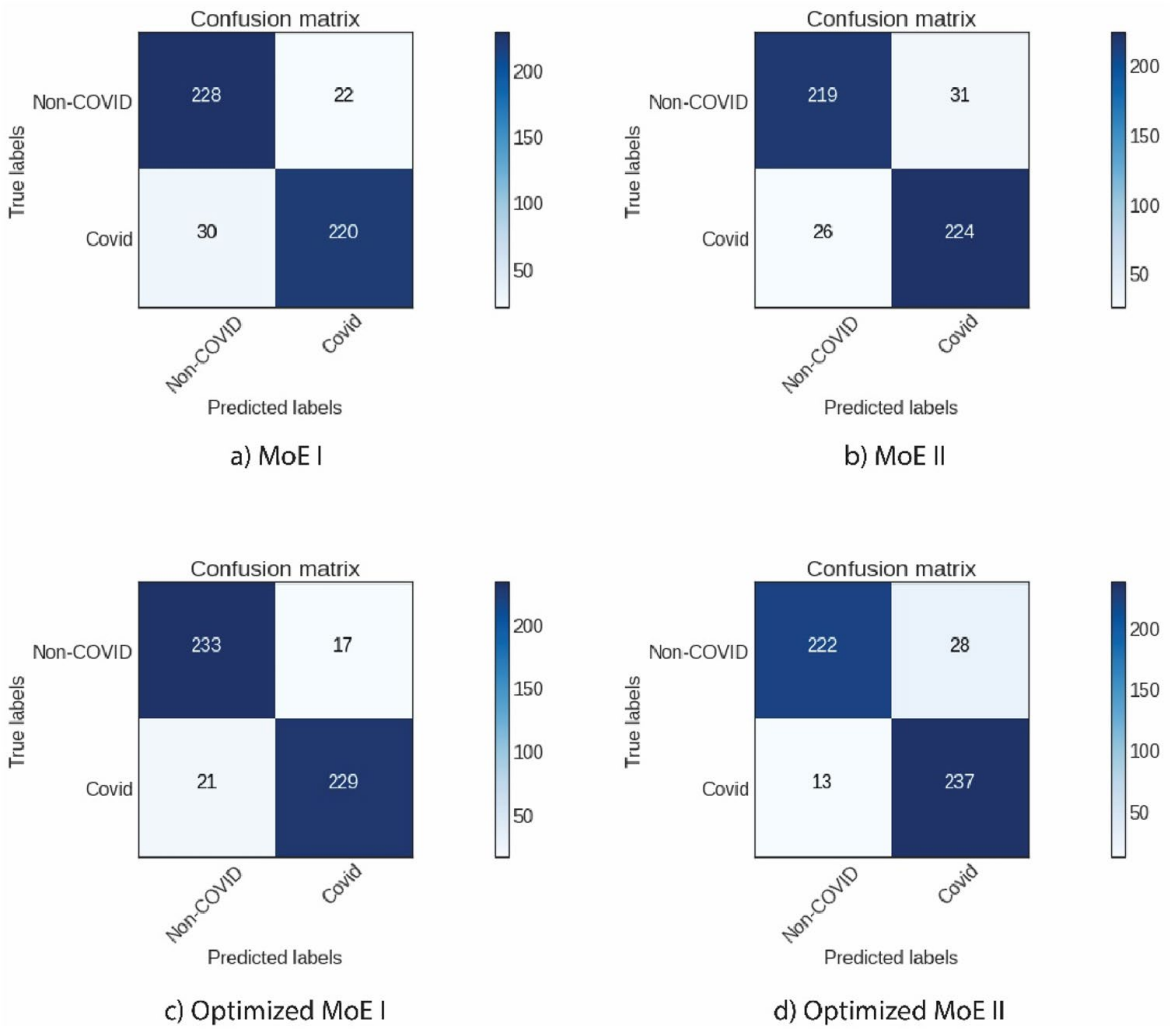
The confusion matrices of the proposed models. (**a**), and (**b**) Show the results for the original MoE I with InceptionV3 as a gating network, and MoE II with InceptionResNetV2 as a gating network. (**c**), and (**d**) Show the results for the Epistocracy optimized version of Moe I and MoE II respectively.

To optimize the overall performance of each mixture of experts, once again, we employed the Epistocracy algorithm to design the architecture and to optimize the hyper-parameters of the classification layers. Figure 9 displays the confusion matrix corresponding to MoE I and MoE II:

As summarized in Table 7, MoE I, and MoE II were able to achieve 93% classification accuracy on non-COVID-19, and 95% accuracy on COVID-19 chest X-rays respectively using the HFHS testing dataset.

**Table 7:** Performance evaluation of MoE I and MoE II.

Mixture of expert	Testing acc.	Classes	Precision	Recall	F1-score
MoE I	0.92	Non-COVID	0.92	**0.93**	0.92
		COVID	0.93	0.92	0.92
MoE II	0.92	Non-COVID	0.94	0.89	0.92
		COVID	0.89	**0.95**	0.92

The final accuracies for detection of COVID-19 and non-COVID-19 infection are displayed in bold.

To classify COVID-19 from CXR images, we developed EpistoNet, a decision tree-based ensemble from MoE I and MoE II. Given a new X-ray image, first MoE II will classify the image. Any image classified by MoE II as COVID-19, is probabilistically 95% accurate. However, if an image was classified as non-COVID-19, MoE I must be consulted. If MoE I classifies the new image as non-COVID-19, we can accept the classification with 93% accuracy. However, if classification by MoE I is COVID-19, this will be considered undetermined where the probability of being COVID-19 would be 7% only.

### Qualitative analysis and visualization

**Figure 10:**
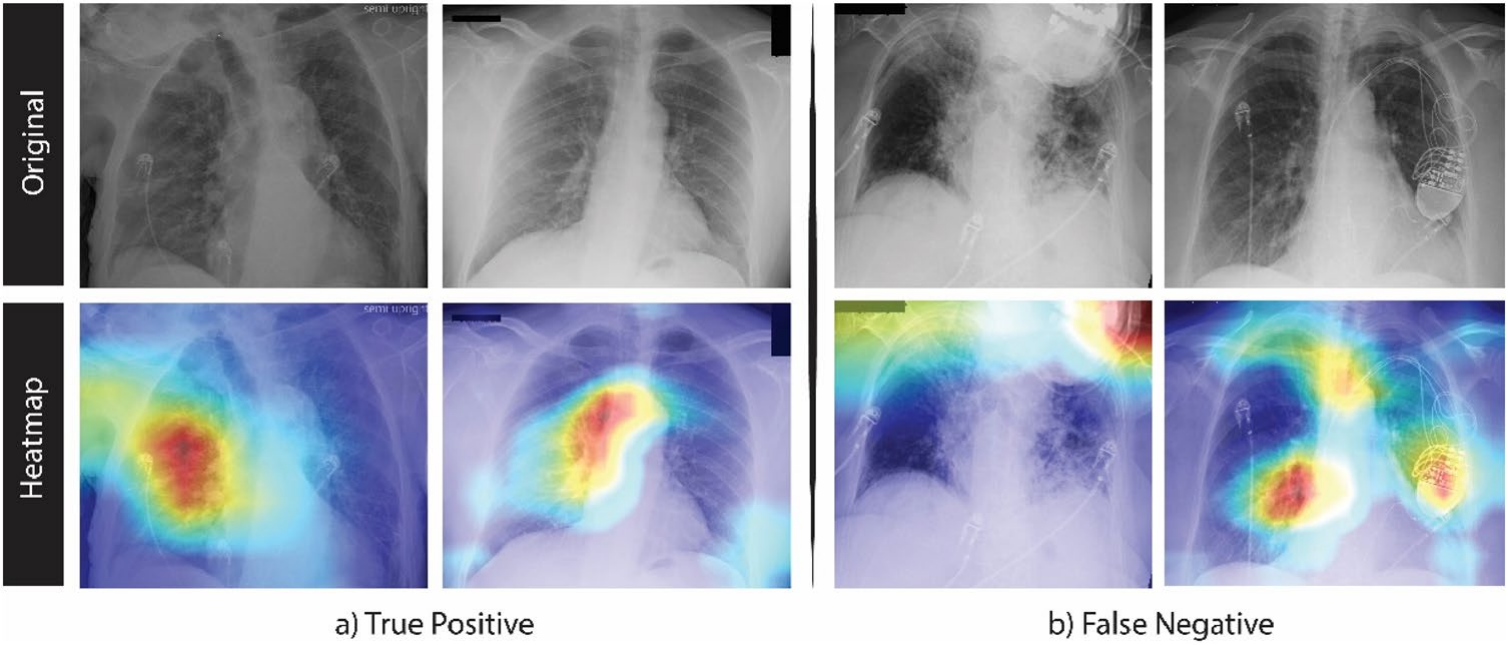
Grad-CAM class activation map of COVID-19: (**a**) True COVID-19 classification, (**b**) false non-COVID-19 classification.

To understand which areas of the image were highlighted and utilized by the model to detect COVID-19 infection, Gradient-weighted Class Activation Mapping^27^ (Grad-CAM) was used. Grad-CAM is a class-discriminative localization map that displays the most relevant and significant regions of an image upon which the classification decision was made by the model. The localization maps are shown as a heatmap, where the intense red color represents the most significant area considered by the classification model. This visualization technique can be used by radiologists and pathologists to localize COVID-19 manifestations in the chest X-rays. In Fig. 10, the Grad-CAM visualization heatmap of some testing images are shown.

From Fig. 10a, it is obvious that our model is focusing on lung opacities, which are the main indicators of COVID-19 infection. In Fig. 10b, the misclassification is due to the indistinguishability of the texture or presence of medical devices and wires in the images which result in very similar probability values for each class.

### EpistoNet performance analysis

Compared to the performance of the individual models using the HFHS testing dataset, EpistoNet exhibits an excellent classification performance. During the initial testing on the Henry Ford dataset before any optimization, the highest accuracy achieved was 86% by VGG16. After partitioning the training-validation dataset into 5 clusters using K-means clustering algorithm and applying the Epistocracy optimization technique and building two mixtures of discriminative experts out of 5 best individual Convolutional Neural Network models the classification accuracy was significantly increased.

Based on experimental results, it is demonstrated that EpistoNet can accurately, and reliably detect COVID-19 infection from X-ray images. The accuracy of the proposed model compared to the related work is quite encouraging, given the limited amount of labeled data and differences in the quality and quantity of samples used for training and testing. Using EpistoNet, the diagnosis of the Coronavirus disease can be done automatically at a low cost, rapidly, and with high accuracy. With isolation of suspicious cases and treatment of infected patients, the spread of the disease can be significantly reduced.

## Conclusion

In this study we proposed EpistoNet, an ensemble of Epistocracy-optimized mixture of discriminative experts for automatic detection of COVID-19 infection from chest X-rays. Each mixture of expert consists of 5 deep convolutional neural networks and a gating network. We evaluated the performance of various state-of-the-art convolutional neural networks using HFHS dataset. Transfer learning was utilized to get a better initialization state for classification of COVID-19 disease. Epistocracy algorithm was also employed to build and optimize the head models composed of neural networks of variable length. The experimental results show that EpistoNet can effectively classify COVID-19 vs. non-COVID-19 infections, even with a limited data set. The accuracy rates achieved by EpistoNet for the classification of COVID-19 were found to be higher than that of stand-alone VGG16 or similar models trained on HFHS dataset. Other approaches lack the generalizability of our method for unseen data due to various pre-processing steps performed and hyper-parameter fine tuning conducted specific to their own dataset. In EpistoNet pipeline, a minimal pre-processing step is required, and Epistocracy algorithm is recruited to systematically optimize the models’ hyper-parameters without any human intervention. EpistoNet can be effectively leveraged as a fast, cheap and portable tool to provide excellent diagnostic aid to healthcare professionals such as physicians and radiologists for the early detection and urgent treatment of patients with COVID-19, mitigating the devastating impact of COVID-19 on lives and livelihoods.
